# Role of NKT Cells in the Pathogenesis of NAFLD

**DOI:** 10.1155/2012/850836

**Published:** 2012-04-05

**Authors:** Kazuto Tajiri, Yukihiro Shimizu

**Affiliations:** ^1^The Third Department of Internal Medicine, Toyama University Hospital, Toyama, Toyama 930-0194, Japan; ^2^Gastroenterology Unit, Takaoka City Hospital, Takaoka, Toyama 933-8550, Japan

## Abstract

Nonalcoholic fatty liver disease (NAFLD) is the most frequent chronic liver disease and shows various inflammatory changes in the liver. Among those inflammatory cells, natural killer T (NKT) cells are found to have a critical role during the disease progression. NKT cells may have a protective role at the early stage with simple steatosis through modification of insulin resistance, whereas they act as a progression factor at the advanced stage with fibrosis. Those processes are thought to depend on interaction between NKT cells and CD1d molecule in the liver.

## 1. Introduction

Nonalcoholic fatty liver disease (NAFLD) is the most frequent chronic liver disease in the world [1]. NAFLD shows various degrees of necroinflammatory changes and fibrosis in the liver and has been shown to lead to cirrhosis and hepatocellular carcinoma (HCC) [2]. As for the progression of NAFLD from simple fatty liver (FL) to nonalcoholic steatohepatitis (NASH), the “two-hit theory,” which proposes the accumulation of fat as the first hit sensitizes the liver to a variety of second “hits” leading to hepatic injury, inflammation and fibrosis, has been generally accepted as an essential mechanism [3], although precise mechanism of the disease progression is still uncertain.

Because various degrees of inflammatory cell infiltration are seen in the livers with NAFLD, especially in NASH, immunological mechanisms are also thought to be profoundly associated with the pathogenesis and progression of NAFLD leading to fibrosis or HCC. However, the precise role of hepatic inflammation or contribution of immune responses in the pathogenesis of NAFLD has not been clarified yet. Recently, innate immune cells including natural killer T (NKT) cells have been shown to contribute to the pathogenesis. In this paper, we summarize and discuss the role of immune reactions in the pathogenesis of NAFLD, especially focusing on NKT cells.

## 2. Hepatic NKT Cells and Their Role in the Pathogenesis of Liver Diseases

### 2.1. NKT Cells in the Liver

The liver contains a unique population of resident mononuclear cells including innate immune cells such as Kupffer cells (KCs) or NKT cells, possibly because of a defense mechanism against constant exposure to a variety of toxins and antigens from intestinal bacteria through portal veins [4]. NKT cells are most abundant in the liver, and their regulatory roles in hepatic inflammation have been reported [5]. NKT cells are the unique subset of cells, which have both T-cell receptor (TCR) and specific surface molecules for natural killer cells [6] and are found with up to 30% of the intrahepatic lymphocytes in mice, and up to 10% of them in humans [7].

### 2.2. Immunoregulatory Role of NKT Cells

NKT cells are divided into type 1 and type 2 NKT cells according to the dependence on the interaction with CD1d, which is a nonpolymorphic glycolipid antigen-presenting molecule structurally related to the class I major histocompatibility complex (MHC). Type 1 NKT cells, which express an invariant TCR containing V*α*14 in mice or V*α*24 in human, recognize glycolipids in conjunction with CD1d, whereas the repertoire of TCR of Type 2 NKT cells are diverse although precise character is less well understood [6]. CD1d is a molecule originally identified on thymocytes or antigen-presenting cells [8, 9]. In normal livers, CD1d is mainly expressed on KCs, but is also expressed on hepatocytes at a very low level. CD1d molecule is upregulated on both hepatocytes and bile duct epithelium in liver diseases including NAFLD [10, 11].

Invariant NKT cells, Type 1 NKT cells, are lipid antigen-specific lymphocytes and produce large amounts of T-helper (Th)1 (e.g., interferon-gamma (IFN-*γ*), tumor necrosis factor-alpha (TNF-*α*)), Th2 (e.g., interleukin (IL)-4, IL-10), and Th17 (e.g., IL-17, IL-22) cytokines through recognition of glycolipid antigen presented on CD1d molecules [12]. Thus, invariant NKT cells may act as immune regulators in various liver diseases. Actually, alterations in the numbers of hepatic NKT cell have been found in various liver diseases such as autoimmune hepatitis, primary biliary cirrhosis, viral hepatitis, or alcoholic and nonalcoholic steatohepatitis. In human autoimmune liver diseases, NKT cells act as proinflammatory cells through hepatocyte apoptosis induced by the release of perforin or granzyme, or by the increased expression of FasL in addition to cytokine production such as IFN-*γ* or TNF-*α* [13]. The cells also play anti-inflammatory functions through Th2 polarization, Th17-dependent mechanisms or regulation of other types of immunoregulatory cells such as regulatory T cells [6, 13, 14]. In murine, a number of studies using concanavalin-A-(ConA-) induced hepatitis or alpha galactosylceramide (*α*-GalCer) induced hepatitis, both of which are murine models of autoimmune hepatitis, have shown the contribution of NKT cells in the disease progression [13, 15]. Collectively, hepatic NKT cells have both proinflammatory and anti-inflammatory functions and play an important regulatory role in the progression of liver diseases.

### 2.3. The Interaction between CD1d and NKT Cells in Lipid Metabolism

On the other hand, the liver has a central role in lipid metabolism with lipolyis, lipogenesis or fat storage. A recent study showed that the function of hepatic NKT cells is rapidly activated by lipids in a CD1d-dependent fashion [16]. Kotas et al. recently demonstrated that CD1d deficiency induces hepatic steatosis and glucose intolerance with high-fat or choline-deficient diet, and glucose intolerance was mainly induced by decreased hepatic insulin sensitivity [17]. Furthermore, CD1d deficiency also led to aggravation of metabolic parameters such as glucose homeostasis and hepatic lipid metabolism [17]. On the other hand, dietary fatty acids can modulate antigen presentation to hepatic NKT cells by a CD1d-dependent manner [18]. CD1d thus can also modulate insulin resistance and play an important role in lipid metabolism, leading to the formation of hepatic inflammation through antigen presentation to NKT cells.

## 3. NKT Cells and NAFLD

As described above, NKT cells modulate hepatic inflammation through CD1d recognition in conjunction with glycolipid antigen. Therefore, it is likely and reasonable to hypothesize that NKT cells contribute to the pathogenesis of NAFLD especially in the formation of hepatic inflammation and disease progression.

### 3.1. The Role of NKT Cells in Animal Models

In murine models, the association between NKT cells and NAFLD has been widely analyzed. Depletion of NKT cells has been reported in ob/ob mice, which are leptin deficient and regarded as a model of obesity-related fatty liver [19, 20]. In ob/ob mice, hepatic sensitization toward proinflammatory conditions is induced by endotoxin from gut, increased production of adipokines, or endoplasmic reticulum (ER) stress, as seen in human NAFLD [19, 21, 22]. Increase in adipokine production or ER stress activates cytokine production from hepatic KCs, especially IL-12, leading to selective depletion of hepatic NKT cells. Recently, Kremer et al. reported that hepatic NKT cells are decreased in hepatosteatosis in KCs- and IL-12-dependent manners [23]. They found that hepatic NKT cells are decreased as hepatosteatosis progresses that is developed by choline-deficient diet, but are preserved in IL-12 deficient mice. Moreover, administration of lipopolysaccharide leads to increase in hepatic IL-12 expression, and the depletion of KCs reduced hepatic IL-12 expression and restored NKT cells. In addition, administration of probiotics has been reported to improve high-fat diet-induced hepatic steatosis and insulin resistance by increasing hepatic NKT cells through reduction in the production of TNF-*α* and nuclear-factor-(NF)-*κ*B-binding activity [24].

Adoptive transfer of NKT cells or treatment with glycolipid antigens has been shown to result in a reduction of hepatic steatosis and improvement of glucose intolerance in ob/ob mice [25, 26]. Moreover, adrenergic activation by norepinephrine injection has been reported to induce expansion of NKT cell population and improve hepatic steatosis [20]. In wild-type mice model fed by choline-deficient diet or high-fat diet, reduction in hepatic NKT cell numbers accompanied by increased Th1 cytokine production has been demonstrated [27, 28]. Those overall data show that hepatic NKT cells are involved in the process of hepatic steatosis through various metabolic factors or cytokines especially produced by KCs.

On the other hand, the role of hepatic NKT cells has not been elucidated during the progression of NAFLD, because neither ob/ob mice nor mice-fed high-fat diet develop significant liver fibrosis. Recent report by Syn et al. demonstrated that hepatic NKT cells are increased in the NAFLD liver partly due to hedgehog pathway activation, leading to promotion of liver fibrosis through activation of hepatic stellate cells (HSCs) [29]. Collectively, murine studies showed that NKT cells seem to act as important players in fat metabolic disorder and have an important role not only in hepatic steatosis (first hit) process but also in disease progression (second hit) in the pathogenesis of NAFLD. 

### 3.2. The Role of NKT Cells in Humans

**Figure 1:**
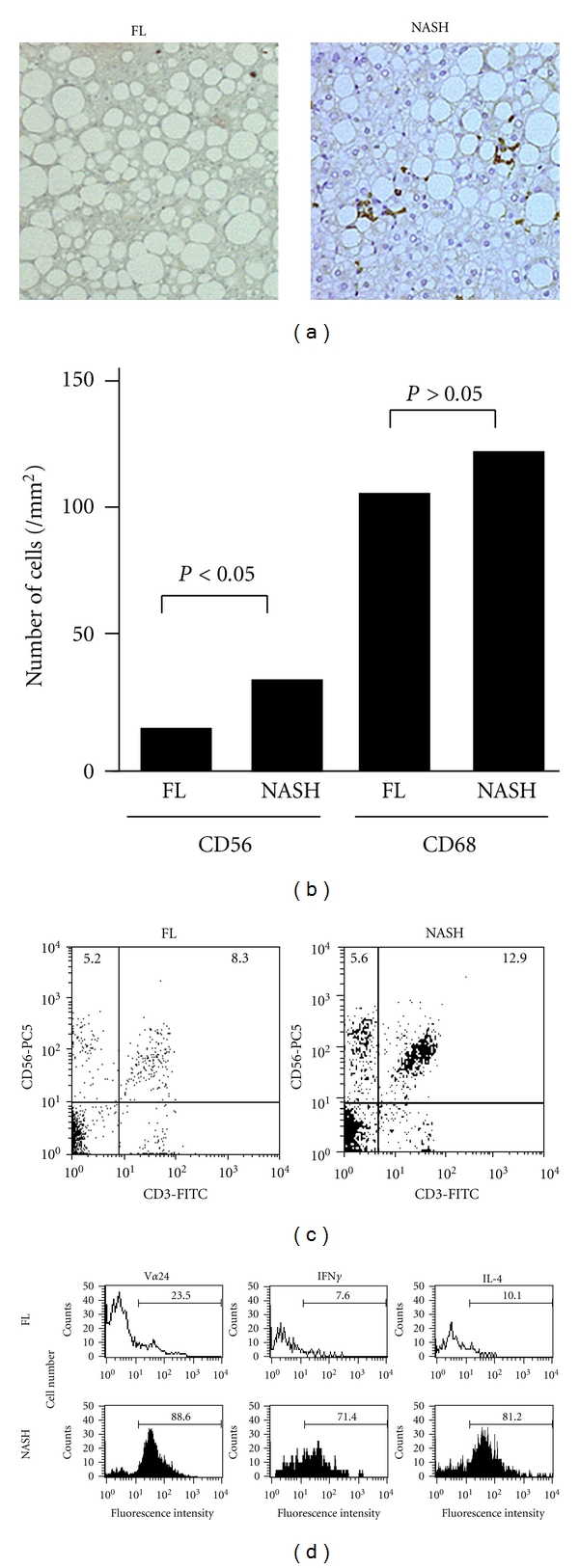
Accumulation of NKT cells in the NAFLD liver with high disease activity. (a) Immunohistochemical study using monoclonal antibody against for CD56 shows accumulation of CD56^+^ cells in the liver of NAFLD with the disease progresses. (FL; fatty liver versus NASH; nonalcoholic steatohepatitis). (b) The number of CD56^+^ or CD68^+^ cells. CD56^+^ cells are significantly increased as the disease progresses. (c) Flow cytometric analysis of isolated intrahepatic mononuclear cells with NAFLD. Numbers in the quadrant represent the percentage of positive cells. Right-upper quadrant represents NKT cells (CD3^+^CD56^+^ cells). (d) Flow cytometric analysis of V*α*24 and intracytoplasmic cytokines of gated CD3^+^CD56^+^ cells among mononuclear cells isolated from livers with NAFLD. Numbers in each histogram represent the percentage of positive cells. These data have been previously presented in [30].

In humans, recent several studies including our report [30] also have provided important findings on the contribution of NKT cells to the pathogenesis of NAFLD. Xu et al. reported that peripheral NKT cells are decreased in NAFLD patients as compared to healthy controls [31], may suggesting preferential recruitment of peripheral NKT cells to the liver. Our group recently analyzed the role of hepatic NKT cells in the pathogenesis of NAFLD using liver biopsy specimens of 54 patients with NAFLD [30]. First, we performed immunohistochemical staining for liver biopsy specimens with NAFLD using monoclonal antibodies against CD56 (NK marker) and CD68 (KCs marker). We found that CD56^+^ cells are increased in the liver with NAFLD as the disease progresses (Figures 1(a) and 1(b)). Then, we analyzed the surface markers and intracytoplasmic cytokines of liver-infiltrating cells isolated from liver biopsy specimens by flow cytometry and found that the number of CD3^+^CD56^+^ NKT cells is increased in NASH as compared with simple FL (Figure 1(c)). Most of those NKT cells express V*α*24, which is the phenotype of invariant NKT cells and produce both Th1 and Th2 cytokines in advanced NAFLD (Figure 1(d)). Furthermore, we demonstrated that antigen-presenting cells such as KCs are more activated and increased the expression of CD1d as the disease progresses [30]. Based on those data, we concluded that hepatic NKT cells could contribute to the disease progression in NAFLD through CD1d recognition. More recently, Syn et al. also reported the accumulation of NKT cells to the liver in progressive NASH accompanied with hedgehog pathway activation [29]. Furthermore, an increase in intrahepatic NKT cell has been shown in the livers with moderate-to-severe steatosis by Adler et al. [32]. On the other hand, Kremer et al. reported a different finding, in which NKT cells are decreased in the liver with NAFLD as steatosis developed. However, those results are from a small sample size with relatively mild NAFLD cases (little steatosis 5 cases, mild steatosis 4 cases, moderate steatosis 3 cases) [23]. Thus, because NKT cells are increased in the livers with human NAFLD at least in cases with advanced stages, they could contribute to the disease progression.

### 3.3. Comparisons of the Role of NKT Cells in the Pathogenesis of NAFLD between Animal Models and Humans

The results from human studies, in which NKT cells contribute to the progression of NAFLD, seem to be inconsistent with those from murine models. In murine models, NKT cells are decreased in the liver with steatosis [19–21, 23, 28] but NKT cells are increased according to the progression of NAFLD in humans [29, 30, 32]. One possible reason in the difference between human and mice is a distinct profile of adipokine production. It has been reported that serum leptin levels are increased in human NASH [33] but not in mice models of NAFLD, and administration of leptin to murine leptin deficiency models, ob/ob mice, actually increases the number of NKT cells [19]. Adipokines such as leptin may thus have a potential role in the regulation of the numbers of intrahepatic NKT cells. Alternatively, investigation on simple steatosis has not fully been done in humans as compared with murine studies, because patients with simple steatosis are usually healthy leading to lack of the opportunity to analyze the disease. Moreover, there has been no murine model for human NASH [34]. Those differences might make the contribution of NKT cells in NAFLD in humans and murine inconsistent. Further investigation for the difference in the role of NKT cells between human and mice need to be done in the future.

### 3.4. Summary and Hypothetical Pathogenesis from Experimental Models (Figure 2)

**Figure 2:**
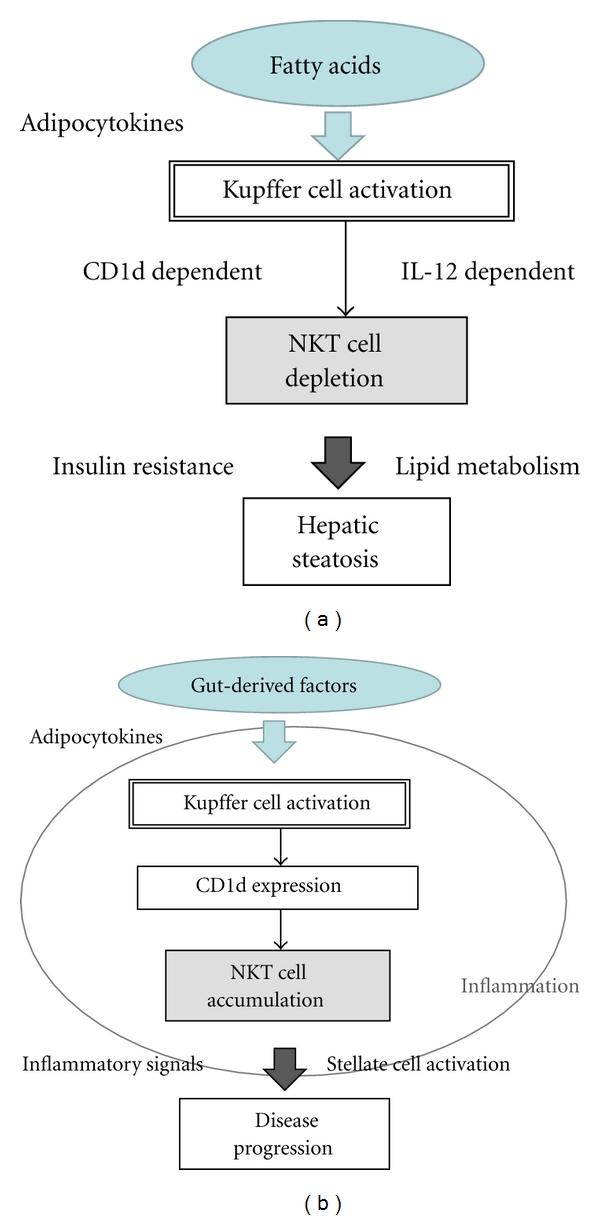
A hypothetical contribution of intrahepatic NKT cells in the progression of NAFLD at the early (a) and the late stage (b). Interaction between NKT cells and CD1d could play an important role in the pathogenesis during the entire phase of NAFLD.

In summary, NKT cells seem to be decreased by activation of KCs through enhanced production of IL-12 at an early stage of NAFLD, whereas those are increased by upregulation of CD1d expression through increased production of adipokines or gut-derived microbiota at an advanced stage of NAFLD in humans. NKT cells may have a protective role at an early stage with simple steatosis by modification of insulin resistance (Figure 2(a)), whereas they act as a progressive factor at an advanced stage with fibrosis through increased proinflammatory cytokine production, NF-*κ*B activation, or HSCs activation (Figure 2(b)). These processes are mainly dependent on interaction of NKT cells with CD1d molecule in the liver. The change in degree or pattern of intrahepatic CD1d expression may thus influence the numbers or functions of NKT cells.

## 4. Conclusion

NKT cells have a regulatory role in the pathogenesis of lipid-metabolic disorder including NAFLD through interaction with CD1d on antigen-presenting cells. Manipulation of NKT cells might thus have a therapeutic potential
